# Spatio-Temporal Projections of the Distribution of the Canopy-Forming Algae *Sargassum* in the Western North Pacific Under Climate Change Scenarios Using the MAXENT Model

**DOI:** 10.3390/biology14060590

**Published:** 2025-05-22

**Authors:** Sun Kyeong Choi, Young Baek Son, Hyun Woo Jeong, Seonggil Go, Sang Rul Park

**Affiliations:** 1 Tropical & Subtropical Research Center, Korea Institute of Ocean Science and Technology, Jeju 63349, Republic of Korea; choisk@kiost.ac.kr (S.K.C.); sonyb@kiost.ac.kr (Y.B.S.); 2 Estuarine & Coastal Ecology Laboratory, Department of Marine Life Sciences, Jeju National University, Jeju 63243, Republic of Korea; hwjeong@stu.jejunu.ac.kr

**Keywords:** climate change, species distribution model, *Sargassum*, macroalgae, marine protected area

## Abstract

Canopy-forming algae play an important role in coastal ecosystems because these species are highly productive and provide habitats and shelter for numerous marine organisms. *Sargassum* is the main genus of canopy-forming algae in the western North Pacific, but despite the importance of their ecological role, studies on the changes in their distribution are still scarce. Based on the present distribution of four *Sargassum* species, this study predicted the geographic distribution of future habitats (2030s, 2060s, and 2090s) under three Shared Socioeconomic Pathway (SSP) scenarios. The environmental variables predicted from the sixth phase of the coupled model intercomparison project (CMIP6) had different impacts depending on the species, with current velocity and water temperature showing high contributions in all four species. According to the projections, three *Sargassum* species (*S. horneri*, *S. macrocarpum*, and *S. patens*) are expected to maintain a higher habitat suitability index (HSI) and suitable habitat (MAXENT ≥ 0.4) through the 2090s under the SSP1-1.9 scenario. However, under the SSP2-4.5 and SSP5-8.5 scenarios, the HSI of the species is projected to gradually decrease in the southern coastal waters of the Korean peninsula and increase in the East Sea (North Korea), with these results intensifying under the SSP5-8.5 scenario. On the other hand, *S. piluliferum* was found to increase its HSI and habitat under the highest emission scenarios. All *Sargassum* species are predicted to shift northward from 0.8° N to 3.8° N by the 2090s under the SSP5-8.5 scenario. Although many marine protected areas exist off the coasts of South Korea and Japan, suitable Sargassum habitats were found to be located within protected reserves between 47.1% and 61.2%, depending on the scenario. These findings on *Sargassum* provide distributional predictions for ecological conservation strategies and provide new evidence for the need for climate change efforts.

## Simple Summary

*Sargassum*, canopy-forming algae, play a key role in coastal ecosystems by supporting marine organisms. This study projects how the habitats of four *Sargassum* species in the western North Pacific may change in the present and future (2030s, 2060s, and 2090s) under three climate scenarios (SSP1-1.9, SSP2-4.5, and SSP5-8.5). Seawater temperature and current velocity were major environmental factors influencing their distribution. Under the low-emission scenario (SSP1-1.9), three species (*S. horneri*, *S. macrocarpum*, and *S. patens*) are expected to maintain suitable habitats until the 2090s, whereas, under higher emissions, their habitats may shrink in the south and shift northward. Conversely, *S. piluliferum* may expand its range under SSP5-8.5. By the 2090s, the four *Sargassum* species are projected to shift northward from 0.8° N to 3.8° N. Although some marine protected areas overlap with these present and future suitable habitats, more conservation efforts are needed due to climate change.

## 1. Introduction

The impacts of climate change on the ocean environment are evident based on various factors, including sea surface temperature, ocean heat content, ocean pH, dissolved oxygen concentration, ocean circulation, sea ice, and sea level [1,2]. Ocean temperature variability due to climate change exhibits different characteristics in various sea regions worldwide [1,3]. In particular, environmental changes are more severe in coastal areas where human activities are frequent [4,5]. Recently, rapid changes in the marine environment have been detected in the western North Pacific. The sea surface temperatures in the East China Sea and the Yellow Sea have rapidly increased due to global warming [4,5,6,7]. In addition, the western North Pacific is increasingly affected by large-scale disturbances, such as typhoons and heavy rainfall, due to the impacts of climate change [8,9], and discharges from the Yangtze River, which contain high levels of nutrients and have low salinity, have increased exponentially [10,11,12]. These changes can have significant impacts on marine ecosystems, particularly marine biodiversity and fisheries [7,13,14]. In particular, benthic organisms that cannot migrate spontaneously may be more vulnerable to environmental changes [15,16].

Climate change can strongly impact coastal ecosystems, driving changes in the survival, growth, and distribution of marine organisms [17,18]. In the rocky benthic area of a temperate or subtropical region, macroalgae have an ecologically important role as primary producers, and large brown algae occupy the benthic ecosystem as major components [19,20]. Among them, *Sargassum* C. Agardh (1820) is the representative genus of the Sargassaceae. They resemble terrestrial trees because they have highly developed structures, including a holdfast, stipe, and blade [21]. Approximately 30 species of *Sargassum* are distributed in South Korea, where they have been reported to occur at various water depths (https://species.nibr.go.kr, accessed on 16 April 2025), from the intertidal zone to below 20 m depths, depending on the species [20,22,23]. *Sargassum* beds exhibit high biomass per unit area because they are composed of dense stands consisting of the same or other *Sargassum* spp., and some species grow to more than 10 m in height [20]. Due to their structural features and biodiversity, *Sargassum* spp. are representative canopy-forming macroalgae on rocky bottoms along the coastal areas of the Korean peninsula and can serve as spawning, breeding, and feeding areas for a variety of shellfish, fish, and other ecologically and economically important marine organisms [24]. However, despite their ecological importance in temperate coastal areas, macroalgae habitats are declining rapidly due to coastal development and anthropogenic changes [25,26,27]. In response, many countries—including the United States, Australia, and Germany—are expanding marine protected areas (MPAs) to conserve critical marine habitats, enhance biodiversity, and promote sustainable fisheries [28]. To improve the effectiveness of these efforts, conservation guidelines and key features have also been developed [29,30]. However, in South Korea, numerous coastal areas, where major benthic communities like macroalgae primarily occur, remain unprotected, despite growing recognition of their ecological importance and vulnerability to climate change (https://www.protectedplanet.net/en/thematic-areas/marine-protected-areas, accessed on 16 April 2025).

Macroalgae habitats are determined by various marine environments, such as water temperature, salinity, light intensity, and nutrient concentrations, which fluctuate with climate change and human activities [17,31]. Macroalgae have distinct distributions due to species-specific preferences for different marine environmental conditions [26,32]. Species unable to adapt to environmental changes will shift their habitat or become extinct [27,31]. This sensitivity of macroalgae to environmental responses makes them potentially useful as biological indicators of climate change [33,34]. By considering the marine environmental characteristics of the habitat based on the past and present occurrences of marine organisms, it is possible to characterize the appropriate environmental conditions for the species’ geographical distribution [25,32,35,36]. Climate change has led to dramatic changes in *Sargassum* forests in the western North Pacific. In Japan, about 1400 ha of *Sargassum* beds disappeared along the Japanese coast between 1978 and 1991, and they have not naturally recovered [27]. In South Korea, barren grounds have significantly increased since large brown algae disappeared, and empty space or crust coralline algae increased [37,38,39].

Marine species distribution data can be utilized to predict the future biogeographic distribution of marine species as their habitats shift in response to climate change [36,40,41]. Because climate change-induced distributional shifts are more likely to occur in marine organisms than in terrestrial organisms, various studies have been conducted to predict range shifts of marine species [32]. Species distribution models (SDMs) can be utilized to predict changes in the distribution of various organisms in coastal ecosystems due to climate change. The Maximum Entropy (MAXENT) model is the most widely used model for predicting species distribution habitats under future scenarios [42]. Species distribution model studies have been conducted for seaweeds to assess habitat suitability under current and future climate scenarios [35,40]. The sixth phase of the coupled model intercomparison project (CMIP6) provides datasets of global temperature change under various scenarios through the end of the 21st century [43], and predicts that high-latitude regions of the Northern Hemisphere will experience more significant changes [44]. Given the severity of climate change impacts and the ecological importance of seaweeds on the Korean peninsula and surrounding waters, predicting changes in their distribution in rocky coastal ecosystems is critical. In particular, understanding how future marine climate change will affect the distribution of each species, depending on its intensity, can provide essential information to better protect and sustain not only seaweeds but also the various marine fisheries resources that depend on them. However, studies of distributional changes for seaweeds in the western North Pacific are lacking [35,41], and no SDM-based habitat suitability assessments have been conducted for seaweeds on the Korean peninsula.

This study aims to assess projected changes in the distribution of *Sargassum* in the western North Pacific under various climate change scenarios and to provide evidence of potential habitat shifts. To understand the impacts of climate change and marine environmental changes on *Sargassum*, this study used the MAXENT model to project the potential distribution of *Sargassum* in the western North Pacific. For four species of *Sargassum*, this study (1) compared historical (pre-2000) and present (post-2000) distributions, (2) quantitatively assessed the impact of environmental variables on species distributions through modeling, and (3) projected the geographical extent of future habitats (2030s, 2060s, and 2090s) under different climate change scenarios. The results can provide important information for the expansion of marine protected areas to conserve marine ecosystems and maintain biodiversity, because *Sargassum* forests are utilized as spawning grounds and nurseries for many fish species in Korean waters.

## 2. Materials and Methods

### 2.1. Study Area

**Figure 1:**
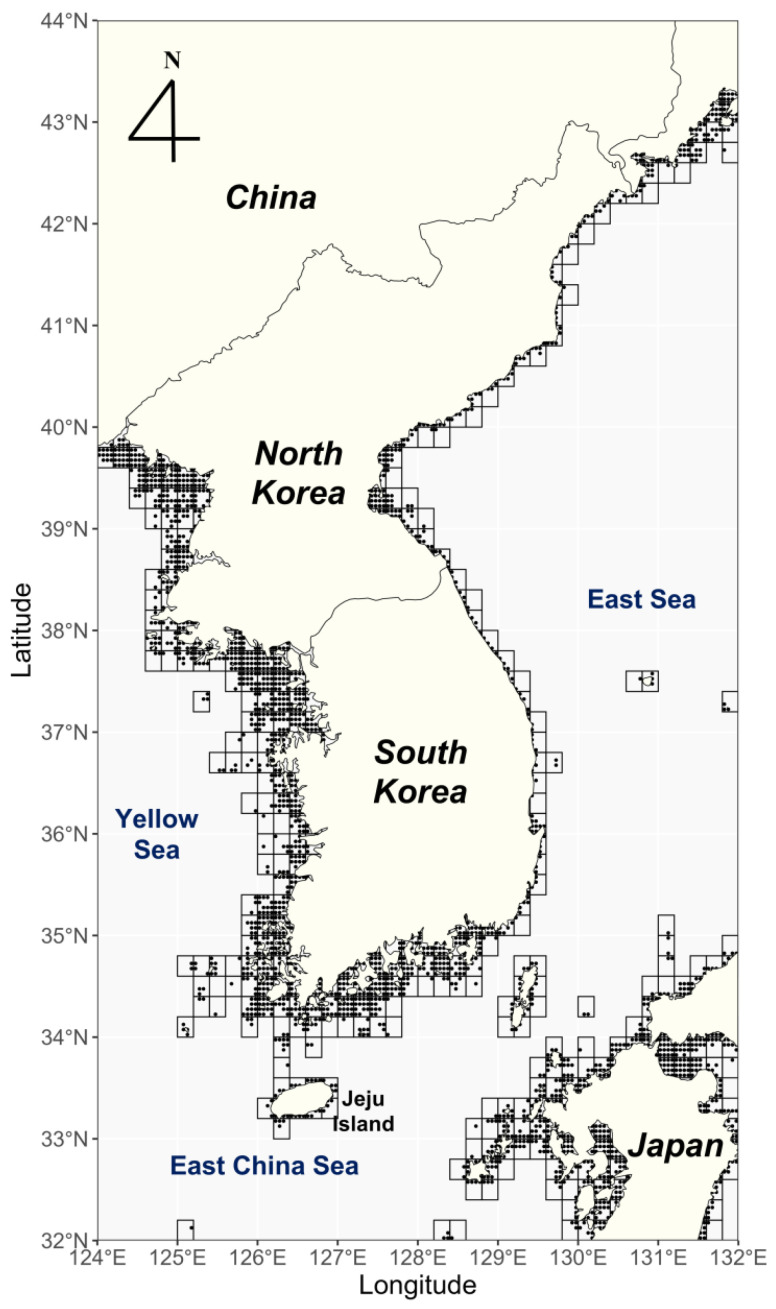
A map of the study area in the western North Pacific. The dots represent the study points, with a resolution of 0.05 degrees utilized in the analysis, and the rectangles represent the base region in this study, averaged over 0.2° × 0.2° intervals.

This study was conducted on the Korean peninsula and part of the southern Japan coast (approximately 32° N to 44° N, 124° E to 132°) located in the western North Pacific of the easternmost part of the Eurasian continent (Figure 1). The southern waters of the Korean peninsula are influenced by the Tsushima Warm Current (a branch of the Kuroshio Current). In contrast, the northern waters of the east coast are influenced by the North Korean Cold Current, resulting in distinct water temperature differences depending on latitude [45]. The Korean peninsula is surrounded by the Yellow Sea to the west, the East China Sea to the south, and the East Sea to the east, exposing it to different marine environments depending on the direction of the sea. It has recently been confirmed that the temperatures of these waters are increasing significantly under the influence of climate change [6,46]. Since the *Sargassum* habitat is reportedly found mainly in the coastal waters, within 20 m depth, we analyzed the area within 20 m depth. The study area in the Yellow Sea was limited to within 5 m depth because the Sea has high turbidity and low water transparency due to its fine sediment characteristics [47,48].

### 2.2. Collection of Data on Sargassum Occurrence

The historical distributions of the four *Sargassum* species were compiled from ‘A catalogue of the seaweeds in Korea’, a review of recorded occurrences of seaweeds in Korea up to 2000 [49]. From these data, 47 distribution records for *Sargassum horneri*, 10 records for *Sargassum macrocarpum*, 26 records for *Sargassum patens*, and 29 records for *Sargassum piluliferum* were identified in South Korea from 1906 to 1999, excluding duplicate records. In addition, we obtained data on the occurrence of each species identified in South Korea from 1989 to 2022 from the Marine Bio-Resource Information System of the National Marine Biodiversity Institute of Korea (https://www.mbris.kr, accessed on 16 April 2025). Only one instance of each collection site information was used, and the occurrence records for each species were 271 for *S. horneri*, 100 for *S. macrocarpum*, 55 for *S. patens*, and 53 for *S. piluliferum*. To compare the distribution characteristics based on the above databases, we divided them into ‘past (pre-2000)’ and ‘present (post-2000)’ based on the centuries, and finally analyzed 67 records for *S. horneri*, 11 records for *S. macrocarpum*, 27 records for *S. patens*, and 33 records for *S. piluliferum* in the past, and 251 records for *S. horneri*, 99 records for *S. macrocarpum*, 54 records for *S. patens*, and 49 records for *S. piluliferum* in the present (Table S1). The occurrence data for *Sargassum* were used a comparison between the past and the present distribution and analysis of latitudinal shifts. In addition, if multiple occurrence records were found within 0.05° × 0.05° intervals in the study area, the data were treated as one and used to model for the estimation and projection of the potential distribution of *Sargassum* under future scenarios.

### 2.3. Environmental Variables

The oceanographic environmental variables known to be associated with the characteristics of macroalgal distribution include water temperature, salinity, current velocity, nitrate concentration, phosphate concentration, pH, and primary productivity, with water temperature and salinity known to be particularly strongly associated with algal growth [32,35,40,41]. Therefore, we selected the mean data of relevant variables that could be related with the distribution of *Sargassum* in their natural environments, and the long-term averages of the maximum and minimum (ltmax and ltmin, respectively) of water temperature and salinity (Table S2). Considering the findings of previous similar studies and the relatively shallow depth of the study area, the variables were derived from surface layer data [32,35,40,41].

The 11 relevant present and future variables were downloaded from Bio-ORACLE v3.0 at a resolution of 0.05 degrees using the ‘biooraler’ package in R, which supports downloading data layers [50]. To determine the optimal environment based on the present distribution of *Sargassum*, we calculated the average of the Bio-ORACLE data in the 2000s and 2010s. To project the potential distribution of *Sargassum* based on future climate conditions, we chose three climate change scenarios, Shared Socioeconomic Pathways (SSP), in the IPCC Sixth Assessment Report (AR6)—SSP1-1.9, SSP2-4.5, and SSP5-8.5—to represent the two extremes and the scenarios in between, and three periods: 2030s, 2060s, and 2090s. The variable data for the present and future were reproduced for the study area (Figure 1).

To avoid overfitting the models to the occurrence data, environmental variables with a relative contribution score of <5% or a correlation of >0.7 with other variables were excluded using the Maximum Entropy (MAXENT) model, with 10 replicates [51]. To select a beta multiplier that would enhance the model’s performance, we varied the beta multiplier, ranging from 1 to 10 by 0.5, and compared the area under the curve (AUC) values, which were used to evaluate the model’s predictive performance. The collinearity of the retained environmental variables with the chosen beta multiplier was assessed using the variance inflation factor (VIF < 5) using the ‘usdm’ package in R statistical software (version 4.4.3) [52].

### 2.4. Species Distribution Modeling and Evaluation of Sargassum

To estimate and project the habitat suitability index (HSI) in the present and future, we applied the MAXENT model, a species distribution model, using the MAXENT v3.4.3 [53]. We randomly selected 70% of the occurrence records in the present (post-2000) for training the MAXENT model, and the remaining 30% were used for testing the model. Then, the MAXENT model was run 10 times for each species under the selected environmental variables and a beta multiplier using the ‘dismo’ package in R [54]. The model’s performance, by species, was evaluated using the projected AUC provided by the receiver operator characteristic (ROC) curve [42], and the true skill statistic (TSS) [55].

To assess the HSI of *Sargassum* in the present and future, we averaged the MAXENT results in 0.2° × 0.2° intervals (Figure 1). We evaluated the HSI at 5 levels: Unsuitability (0–0.2), Low suitability (0.2–0.4), Moderate suitability (0.4–0.6), High suitability (0.6–0.8), and Optimal suitability (0.8–1.0), based on the average MAXENT. We defined the Moderate, High, and Optimal suitable HSIs as suitable habitats for *Sargassum* (average MAXENT ≥ 0.4), and the Unsuitability and Low suitability HSIs as unsuitable habitats for *Sargassum* (average MAXENT < 0.4) to calculate suitable habitats in the present and future. The suitable habitat for each scenario was calculated by adding the suitable habitat area, which was calculated as a function of latitude.

We determined changes in the HSI for *Sargassum* under the future scenarios compared to the present, defining four categories: Absence (unsuitable habitat in the present and future), Constriction (changes from suitable to unsuitable), Expansion (changes from unsuitable to suitable), and Stability (suitable in both periods). Furthermore, we considered the latitudinal shifts for each climate change scenario by calculating an average of latitudes weighted by the MAXENT results, and the species richness by examining the number of suitable habitats for each species at the location to understand how climate change affects the hotspot distributions of four *Sargassum* species. We also considered whether potential habitats for *Sargassum* are included in the MPA. The potential habitat was determined based on the absence or presence of species richness. We defined ‘Absence’ as an unsuitable habitat for all species, ‘Unprotected’ as a suitable habitat for one or more *Sargassum* species, excluding the MPA, and ‘Protected’ as a suitable area for one or more *Sargassum* species, including the MPA.

## 3. Results

### 3.1. Past and Present Distribution of Sargassum in South Korea

**Figure 2:**
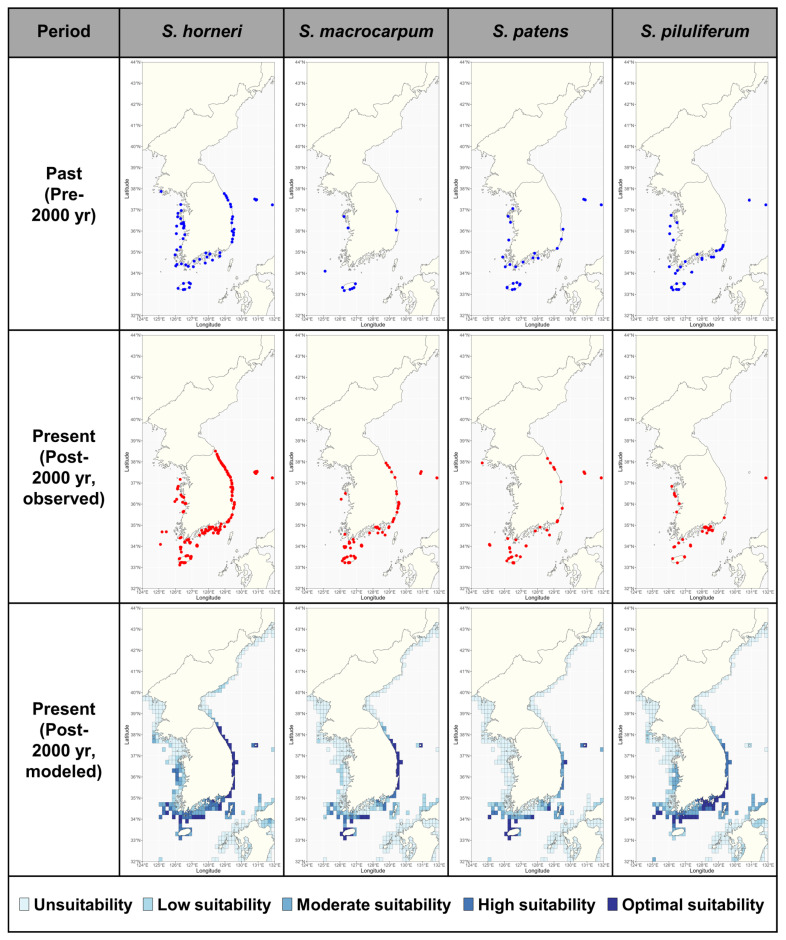
The distribution of *Sargassum* in South Korea based on historical (pre-2000) and present (post-2000) records, and the distribution of the estimated habitat suitability index (HSI) of *Sargassum* based on the Maximum Entropy (MAXENT) model for the present period (2000–2020). The HSIs are defined by MAXENT, which assigns a MAXENT result of 0–0.2 to a HSI of ‘Unsuitability’, 0.2–0.4 to ‘Low suitability’, 0.4–0.6 to ‘Moderate suitability’, 0.6–0.8 to ‘High suitability’, and 0.8–1 to ‘Optimal suitability’.

The distribution of *Sargassum* by century showed different patterns for different species in South Korea (Figure 2). The comparison of distribution maps showed that *Sargassum horneri* had a wider distribution after 2000, especially in the East Sea, where it expanded its range North of 38° N. *Sargassum macrocarpum* was found in Jeju Island, the Yellow Sea, and parts of the East Sea before 2000. However, after 2000, it was found along Jeju Island, the southern coast of the Korean peninsula, and along the entire East Sea. In the past, *Sargassum patens* and *Sargassum piluliferum* showed similar distribution patterns, with little occurrence in the East Sea. After 2000, *S. patens* was confirmed as far north as the East Sea, while *S. piluliferum* did not show significant changes, indicating different distribution patterns.

### 3.2. Optimal Conditions and Performance of the MAXENT Model

The oceanographic environmental variable data for the present and future were obtained and reproduced for the study area. Species-specific models with the highest AUC values were constructed using selected combinations of three or four uncorrelated environmental variables and optimized beta multipliers (Table 1; Figure S1). For *S. horneri*, the optimal model (highest AUC) was built using a beta multiplier of 2.5 and included the following variables: ltmin of water temperature, mean of current velocity, ltmax of water temperature, and mean salinity, with respective contributions of 39.45%, 35.94%, 15.18%, and 9.43% (Table 2). The corresponding VIFs were 4.38, 1.15, 3.22, and 2.46, indicating no strong multicollinearity. The best model for *S. macrocarpum* used a beta multiplier of 1 and included the mean of current velocity (contribution 55.28%, VIF 1.10), ltmin of water temperature (26.24%, 2.61), ltmax of water temperature (10.79%, 2.19), and mean primary productivity (7.69%, 1.48). For *S. patens*, the model with the highest AUC was constructed with a beta multiplier of 1, incorporating the mean of current velocity (39.17%, 1.04), mean of water temperature (36.61%, 1.09), and mean of primary productivity (24.22%, 1.07). Lastly, the optimal model for *S. piluliferum* was obtained using a beta multiplier of 1.5, with the selected variables being the mean of current velocity (48.75%, 1.05), ltmin of water temperature (27.04%, 1.52), mean of nitrate concentration (14.91%, 1.29), and ltmin of salinity (9.30%, 1.23).

### 3.3. Analysis of Habitat Suitability for Sargassum

**Figure 3:**
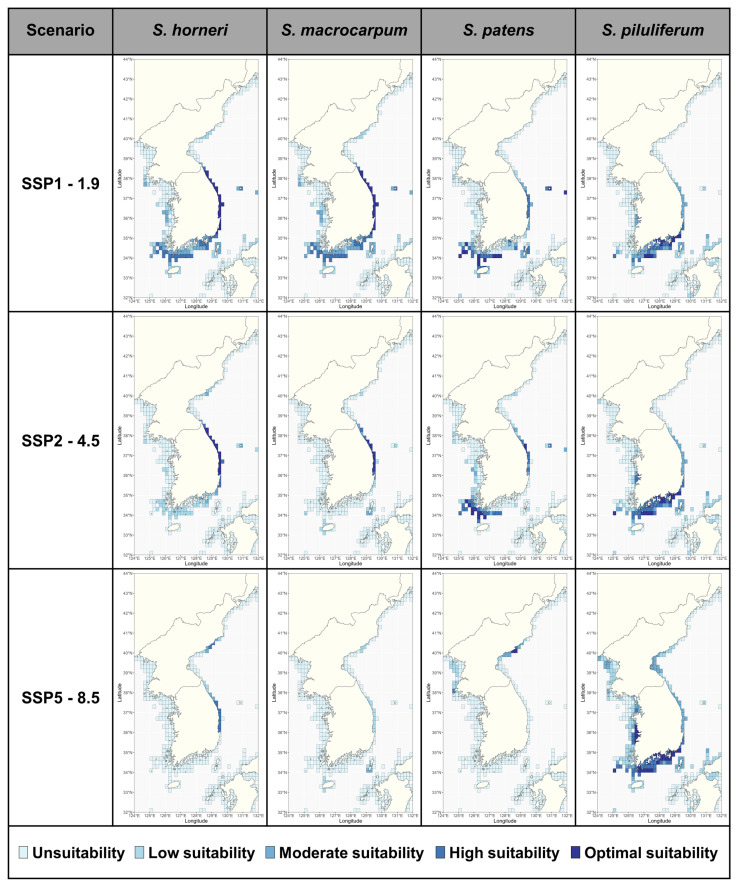
The distribution of the projected habitat suitability index (HSI) of *Sargassum* based on the Maximum Entropy (MAXENT) model for the 2090s (2090–2100). The HSIs are defined by MAXENT, which assigns a MAXENT result of 0–0.2 to a HSI of ’Unsuitability’, 0.2–0.4 to ’Low suitability’, 0.4–0.6 to ’Moderate suitability’, 0.6–0.8 to ’High suitability’, and 0.8–1 to ’Optimal suitability’.

The present habitat suitability indexes (HSIs) for the four *Sargassum* species estimated using the MAXENT model showed similar distributions to the recorded habitat distributions (Figure 2). The HSIs within the study area varied by species, with *S. horneri* having the highest and *S. patens* having the lowest (Figure 2). Future HSIs under climate change scenarios were projected to undergo significant species-specific changes (Figure 3; Figure S2; Figure S3). According to the projections, *S. horneri* is currently expected to have the highest HSI in coastal waters of the East Sea; this result was maintained through to the 2090s under the SSP1-1.9 scenario. However, under the SSP2-4.5 and SSP5-8.5 scenarios, the HSI of the species was projected to gradually decrease in the southern coastal waters and increase in the East Sea (North Korea) coastal waters, with these results intensifying under the SSP5-8.5 scenario. *Sargassum macrocarpum* and *S. patens* were estimated to have similar distributions in the present (Figure 2). *Sargassum macrocarpum* maintained the HSI in the East Sea under both the SSP1-1.9 and the SSP2-4.5 scenarios but experienced the most severe changes in its future distribution in the 2090s under the SSP5-8.5 scenario, with all moderately suitable areas in the Korean peninsula predicted to disappear (Figure 3; Figure S2; Figure S3). *Sargassum patens* maintained a HSI in the Yellow Sea, Jeju Island, and parts of the East Sea until the 2090s under the SSP1-1.9 and SSP2-4.5 scenarios (Figure 3; Figure S2; Figure S3). In the SSP5-8.5 scenario, the HSI of the species is predicted to decrease along all coasts of South Korea while increasing in the East Sea (North Korea). *Sargassum piluliferum* was projected to have a different future distribution than the other three species of *Sargassum*, with a predicted decrease in its HSI under the highest emissions scenario (Figure 3; Figure S2; Figure S3).

### 3.4. Potential Suitable Habitats for Sargassum

**Figure 4:**
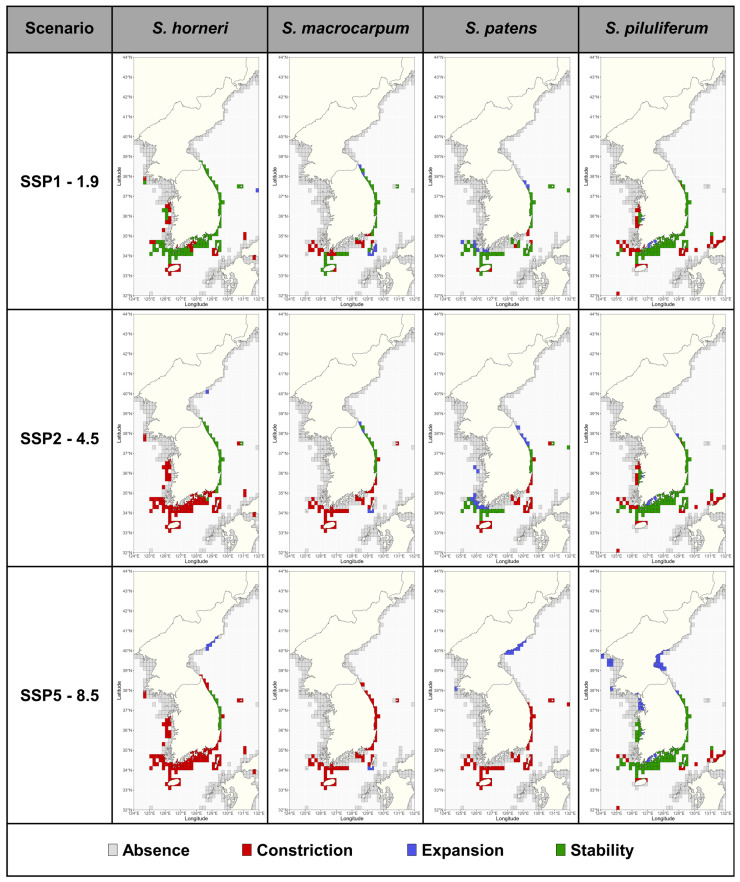
The changes in suitable habitats for *Sargassum* under the climate change scenarios in the 2090s (2090–2100) compared to the present (2000–2020). ‘Absence’ means unsuitable habitat (MAXENT < 0.4) in the present and future, ‘Constriction’ means a change from a suitable habitat (MAXENT ≥ 0.4) to an unsuitable habitat, ‘Expansion’ means a change from an unsuitable habitat to a suitable habitat, and ‘Stability’ means the habitat is suitable in both periods.

At present, the total suitable habitat (average MAXENT ≥ 0.4) for the four species of *Sargassum* covers 16,917 km^2^ and is mainly concentrated in Jeju Island, the southern coast of the Korean peninsula, and the East Sea of South Korea (Figure 2; Table 3). The suitable area for *Sargassum* showed species-specific differences, being higher in *S. horneri* and *S. piluliferum* and lower in *S. macrocarpum* and *S. patens* (Table 3). Future projections indicated that the potentially suitable habitat for *Sargassum* was predicted to change in distribution under three climate change scenarios (Figure 4; Figure S4; Figure S5; Table 3). Under the SSP1-1.9 scenario, all four *Sargassum* species were predicted to lose habitats in some waters along the southern coast of the Korean peninsula, while maintaining habitats in most other areas (Figure 2). The suitable area was projected to fluctuate between +9.9% and −34.4% by the 2030s, depending on the species, following a similar pattern through to the 2090s (Table 3). Under the SSP2-4.5 and SSP5-8.5 scenarios, all *Sargassum* species showed intensification trends with time and emission scenario severity, apart from *S. piluliferum* (Figure 4; Figure S4; Figure S5; Table 3). In particular, under the SSP5-8.5 scenario, suitable habits for these three species were projected to decrease by more than 84% by the 2090s, with some expansion of suitable habitats in the East Sea (North Korea). On the other hand, *S. piluliferum*’s habitat was found to increase under the SSP5-8.5 scenario; as a result, it was predicted that its total suitable habitat will increase in the 2090s, despite the extreme reduction in the habitats of the other three species (Table 3).

### 3.5. Latitudinal Centroid Shift and Community Changes Under Climate Change

**Figure 5:**
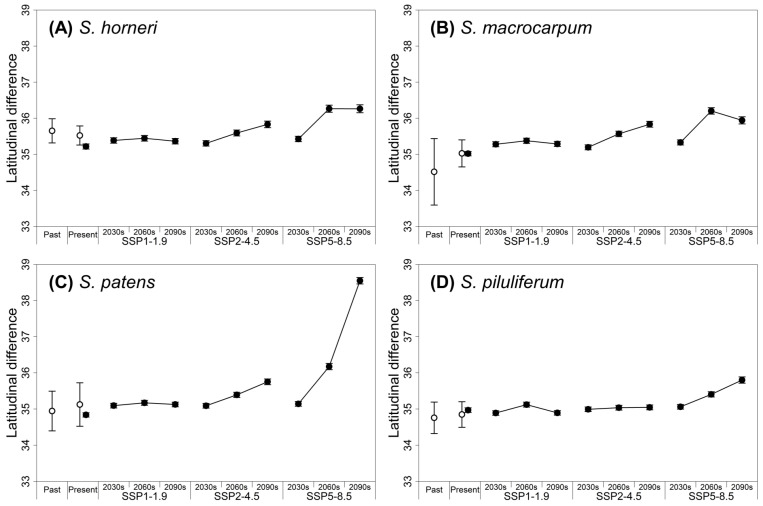
The latitudinal centroid variation of *Sargassum* based on the recorded data (empty circle) and projected model (filled circle) under climate change scenarios from the past (pre-2000) to the 2090s (2090–2100). (**A**) *Sargassum horneri*; (**B**) *Sargassum macrocarpum*; (**C**) *Sargassum patens*; (**D**) *Sargassum piluliferum*.

Based on the observed distribution, the latitudinal centroid of the present habitat of *Sargassum* was slightly elevated compared to the past based on species; however, this change was not significantly different (Figure 5). The current latitudinal centroids based on the MAXENT of the four *Sargassum* species were located from 34.8° N to 35.2° N, consistent with the present centroid from the recorded distribution by species (Figure 5). From the 2030s to the 2090s, the center of distribution was projected to shift northward, with considerable variation across species and climate change scenarios. Under the SSP1-1.9 scenario, the centroid of all four species was predicted to shift northward by less than +0.4° N until the 2090s. In contrast, under the SSP5-8.5 scenario, all species were predicted to shift northward by more than +0.8°N, with *S. patens*, in particular, experiencing a centroid shift to 38.6° N (+3.8° N) by the 2090s.

**Figure 6:**
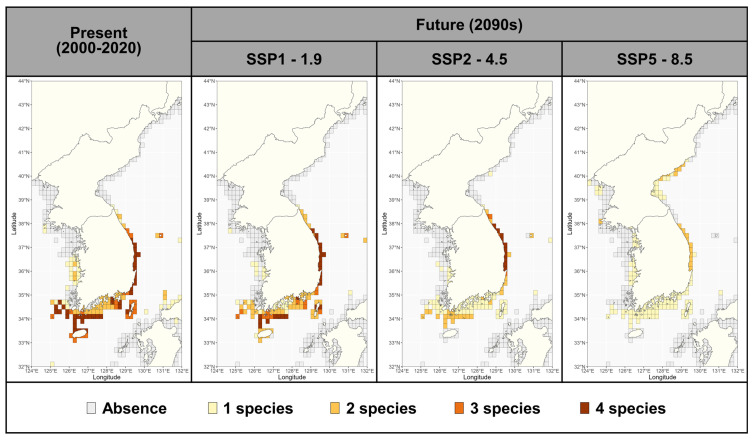
The richness of *Sargassum* under current (2000–2020) and future (2090–2100) scenarios. The numbers represent the number of species that deem the habitat suitable (MAXENT ≥ 0.4), and ‘Absence’ means that all species deem the habitat unsuitable.

In the present period, the four *Sargassum* species are mainly found in the East Sea (South Korea) and Jeju waters (Figure 6). Under the SSP1-1.9 scenario, the abundance of *Sargassum* was maintained in the East Sea until the 2090s, with some declines in the southern waters of the Korean peninsula (Figure 6; Figure S6). The richness of *Sargassum* in the East Sea was maintained until the 2090s under the SSP2-4.5 scenarios but decreased in the southern waters of the Korean peninsula, especially in Jeju waters, which became unsuitable areas (Figure 6). Under the SSP5-8.5 scenario, the species abundance was predicted to decrease in all habitats in South Korea, with one or fewer species predicted to be present in all study waters except the East Sea, while the distribution of *Sargassum* was shown to expand in the East Sea (North Korea).

## 4. Discussion

### 4.1. Environmental Influence on Sargassum Distribution

The growth and distribution of seaweeds are determined by various physicochemical environmental factors [17,31]. In this study, water temperature, salinity, current velocity, primary productivity, and nitrate concentration were identified as environmental variables that determined the distribution of *Sargassum* species. In particular, seawater temperature and current velocity showed high contributions to the distributions of all four species (Table 1). Seawater temperature influenced the prediction of *Sargassum* distribution patterns, with the ltmin and ltmax reflecting extreme temperatures, depending on the species. Our study suggests that the optimal seawater temperature range for the *Sargassum* species we examined is between 6 and 24 °C (Figure S1). The mid-latitude waters of the western North Pacific, where the study area is located, are predicted to experience more rapid seawater temperature increases under climate change [56]. Ltmin, which reflects winter seawater temperatures, is associated with the expansion of the northern range in the East Sea (North Korea), while ltmax, which reflects summer seawater temperatures, can determine the habitat boundary of the southern range in Jeju waters. Under the high emission scenario, the distribution of *Sargassum* was also predicted to change significantly at the southern and northern boundaries of its range. This finding is supported by other studies of marine organisms based on model projections of temperate waters [35,56].

In this study, current velocity was also found to be an important variable in the change in *Sargassum* habitat suitability, with the response curves for seawater flow rate predicting an increase in habitat suitability for these species with increasing flow rates within a range of 0.7 m s^−1^ (Figure S1). These results are consistent with previous findings, which showed that a certain level of increased flow velocity is positively correlated with the growth and distribution of canopy-forming algae [57,58]. Although *Sargassum* would be at a spatial disadvantage compared with fast-growing annual macroalgae, they can survive in waters with high current velocities because their holdfasts are broadly formed like the roots of terrestrial plants, and their main branches are soft and elongated, giving them morphological characteristics that allow them to respond flexibly to currents [24,58]. Moreover, higher water flow velocities allow macroalgae to absorb more of the nutrients that are continuously supplied along the flowing water [59,60].

Based on our model, salinity, primary productivity, and nitrate concentration were also influential environmental variables (Table 1). Low salinity affects the distribution of *Sargassum* in the Yellow Sea and the East China Sea [24,61]. In this study, we projected that *Sargassum* would be distributed at salinities of > 30, and the low-salinity plume impacted by the diluted water from the Yangtze River spread widely into the East China Sea and the Yellow Sea [12], suggesting that salinity is a strong environmental driver of *Sargassum* distribution. The high correlation between primary productivity and habitat suitability for *Sargassum* in this study is consistent with previous studies of canopy-forming algae, including *S. horneri*, where primary productivity was found to be an important environmental variable determining the distribution of these species [35,62]. Primary productivity as a bio-geochemical variable is associated with marine environmental variables, such as light, water transparency, and the concentration of nitrogen available to algae, which directly affect the population dynamics of *Sargassum* [24,63,64].

### 4.2. Habitat Shifts and Changes in the Past, Present, and Future

In this study, the present distribution of *Sargassum* was found to have expanded northward into the waters of the East Sea when compared with the past, whereas the centroid shift analysis did not show a significant difference (Figure 5). This is due to the rapid increase in winter water temperatures in the study area from the past to the present [6,65], which has resulted in the expansion of its habitat in the north; however, the main *Sargassum* habitats, the waters off Jeju Island and the southern part of the Korean peninsula, are still well maintained. The future suitable habitats for *Sargassum* were predicted to vary significantly under different climate change scenarios, especially under the SSP5-8.5 scenario (Figure 4), consistent with previous studies showing that species distribution ranges and suitable habitats expand or contract at habitat extremes due to climate change [35,40,41]. In this study, we projected that suitable habitats for *Sargassum* will decrease in most of the southern coast of South Korea and expand in North Korean waters above 39° N. These results led to the prediction that the center of *Sargassum* distribution would shift northward. These results suggest that habitat variability at both edges could result in a dramatic reduction in suitable habitats for the species in the South Korean waters. Despite this variability, the suitable habitat for *S. piluliferum* was predicted to increase under the SSP5-8.5 scenario. For this species, salinity (ltmin) and nitrate were identified as valid environmental variables that differ from those of other species and contribute to increased habitat suitability along the east coast and south coast of the Korean peninsula under the SSP5-8.5 scenario.

### 4.3. Ecological and Conservation Implications for Sargassum

The suitable habitats for the four *Sargassum* species showed two patterns under different climate change scenarios. *Sargassum piluliferum* was predicted to increase under the highest emissions scenario. This species is found in relatively shallow waters within a 5 m depth [66]. In contrast, the other three species predicted to decrease in distribution occur at depths ranging from 0 to 20 m [20,66,67]. The richness of the *Sargassum* forest was predicted to decrease depending on the climate change scenario. This result is consistent with those of other studies, which showed that the diversity of canopy-forming algae is decreasing in the western North Pacific [27,35,38]. These results also suggest that the abundance of the *Sargassum* forest may vary with depth due to differences in species-specific habitat depths, suggesting that *S. piluliferum* may play an important role under SSP5-8.5 conditions. These differences in species-specific distributions under various climate change scenarios emphasize that projecting habitat suitability at the species level rather than the genus level can predict changes in regional-scale *Sargassum* forests.

**Figure 7:**
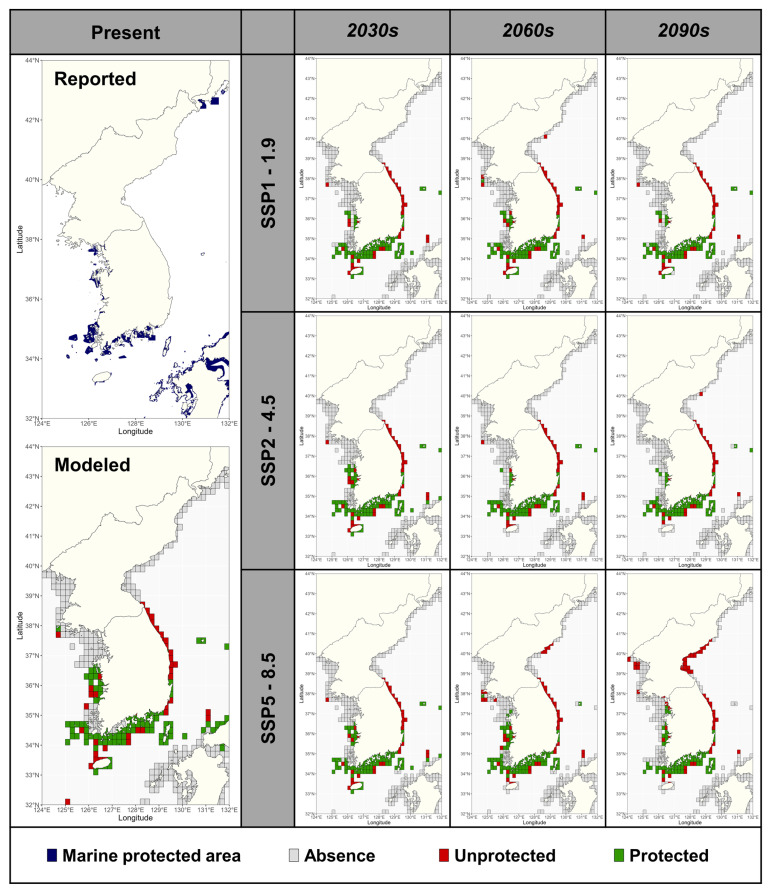
Marine protected areas (MPAs) in the study area (reported) and the protected distribution of *Sargassum* based on different scenarios. ‘Absence’ represents unsuitable habitats (MAXENT ≥ 0.4) for all species, ‘Unprotected’ represents suitable habitats for one or more *Sargassum* species but excludes MPAs, and ‘Protected’ represents suitable areas for one or more species and includes MPAs.

The Kunming–Montreal Global Biodiversity Framework (KM-GBF), approved by the Fifteenth Conference of the Parties (COP15) of the Convention on Biological Diversity (CBD), has the flagship target of ‘30 × 30’, protecting 30% of land, waters, and seas in a representative way by 2030 (https://www.cbd.int/gbf/targets, accessed on 16 April 2025). According to the World Database of Protected Areas (WDPAs), MPAs in South Korea cover an area of 7771 km^2^, and only 2.24% of the total area of the Korean waters (https://www.protectedplanet.net/en/thematic-areas/marine-protected-areas, accessed on 16 April 2025). For this study, we converted the MPAs covering Korea, Japan, and Russia in the study area at a 0.05-degree resolution, and the area identified was approximately 29,102 km^2^ (Figure 7). Overlaying the distribution of suitable *Sargassum* habitats with MPAs, we found that 62.4% of the suitable habitat is currently located within MPAs, whereas 47.1% of the suitable habitats in the 2090s under SSP5-8.5 will be contained within MPAs (Figure 7). Given the resolution of our study, it is likely that the *Sargassum* forest area contained within protected areas is smaller, with larger suitable habitats remaining unprotected. In Japan, 13.79% of the Japanese marine area is designated as MPAs, and most of the coastal areas are included in marine reserves (https://www.protectedplanet.net/en/thematic-areas/marine-protected-areas, accessed on 16 April 2025); thus, most of the suitable *Sargassum* habitats in this study are located in MPAs (Figure 7). However, based on this study, the East Sea and Jeju waters in South Korea are projected to see the most extreme fluctuations in *Sargassum* forests, although most coastal areas are excluded from the MPAs. The *Sargassum* studied here consists mostly of species endemic to the western North Pacific (https://www.algaebase.org, accessed on 16 April 2025), and may become endangered under climate change scenarios; thus, it is necessary to develop conservation strategies. By projecting the response of *Sargassum* forests to climate change, this study provides evidence of the need to mitigate the rate of ocean warming; moreover, these results can be used as a basis for designating regional MPAs based on the importance of marine biodiversity and marine ecosystems.

## 5. Conclusions

This study demonstrates that the future distribution of four *Sargassum* species around the Korean peninsula will be strongly influenced by environmental variables, particularly seawater temperature and current velocity. The species-specific responses to climate change scenarios revealed distinct patterns: while the suitable habitat for *S. piluliferum* may expand under the SSP5-8.5 scenario, the other three species are expected to experience reductions in habitat suitability, particularly in southern coastal regions. These changes will be accompanied by a projected northward shift in distribution, highlighting the potential restructuring of coastal algal communities in response to ocean warming. Importantly, our analysis shows that a significant portion of future suitable habitats for *Sargassum* may fall outside existing MPAs, especially in South Korean waters, where distributional shifts are expected to be most pronounced. These findings underscore the need to enhance marine conservation strategies by incorporating species-specific predictions and expanding the coverage of protected areas. As *Sargassum* forests play a key ecological role in coastal ecosystems, targeted conservation efforts will be critical to preserving biodiversity and supporting ecosystem resilience under changing climate conditions.

## Figures and Tables

**Table 1:** Selected variables, contribution, and variance inflation factor (VIF) by species, as determined using the MAXENT model under each beta multiplier.

Variable	Evaluation	*S. horneri*	*S. macrocarpum*	*S. patens*	*S. piluliferum*
	Beta multiplier	2.5	1.0	1.0	1.5
Ocean temperature (Ltmax)	Contribution	15.18	10.79	-	-
	VIF	3.22	2.19	-	-
Ocean temperature (Ltmin)	Contribution	39.45	26.24	-	27.04
	VIF	4.38	2.61	-	1.52
Ocean temperature (Mean)	Contribution	-	-	36.61	-
	VIF	-	-	1.09	-
Salinity (Ltmin)	Contribution	-	-	-	9.30
	VIF	-	-	-	1.23
Salinity (Ltmax)	Contribution	9.43	-	-	-
	VIF	2.46	-	-	-
Sea water velocity (Mean)	Contribution	35.94	55.28	39.17	48.75
	VIF	1.15	1.10	1.04	1.05
Primary productivity (Mean)	Contribution	-	7.69	36.61	-
	VIF	-	1.48	1.09	-
Nitrate (Mean)	Contribution	-	-	-	14.91
	VIF	-	-	-	1.29

**Table 2:** The evaluated models’ algorithm performance (the projected area under the curve (AUC) and the true skill statistic (TSS) by species, with the selected oceanographic environmental variables and the beta multiplier.

Species	AUC	TSS
*S. horneri*	0.8894 ± 0.0212	0.6330 ± 0.0663
*S. macrocarpum*	0.9110 ± 0.0175	0.6990 ± 0.0597
*S. patens*	0.9024 ± 0.0468	0.7256 ± 0.0860
*S. piluliferum*	0.8498 ± 0.0465	0.5594 ± 0.0957

**Table 3:** Changes in suitable habitats for *Sargassum* (compared with the present) under different climate scenarios.

Scenarios		*S. horneri*		*S. macrocarpum*		*S. patens*		*S. piluliferum*		Total	
		Area (×10^3^ km^2^)	Trend (%)	Area (×10^3^ km^2^)	Trend (%)	Area (×10^3^ km^2^)	Trend (%)	Area (×10^3^ km^2^)	Trend (%)	Area (×10^3^ km^2^)	Trend (%)
Present		14.40	-	6.61	-	5.61	-	13.85	-	16.92	-
SSP1-1.9	2030s	10.33	−28.25	4.34	−34.42	6.17	+9.91	10.74	−22.49	14.12	−16.5
	2060s	9.81	−31.87	3.69	−44.12	5.93	+5.78	12.04	−13.09	15.08	−10.8
	2090s	10.21	−29.12	4.44	−32.89	6.26	+11.66	9.73	−29.80	13.69	−19.0
SSP2-4.5	2030s	12.40	−13.91	5.46	−17.49	6.29	+12.14	13.04	−5.78	15.83	−6.4
	2060s	6.80	−52.81	2.14	−67.61	4.61	−17.87	12.15	−12.27	14.71	−13.0
	2090s	2.77	−80.78	1.45	−78.00	4.69	−16.32	11.37	−17.94	14.11	−16.5
SSP5-8.5	2030s	9.62	−33.18	3.62	−45.20	5.25	−6.51	14.12	+1.93	15.62	−7.6
	2060s	2.37	−83.57	1.48	−77.63	4.28	−23.70	16.98	+22.58	18.06	+6.7
	2090s	1.52	−89.42	0.10	−98.45	0.90	−83.96	18.19	+31.29	19.07	+12.5

## Data Availability

Data are contained within the article and Supplementary Materials.

## Supplementary Materials

The following supporting information can be downloaded at https://www.mdpi.com/article/10.3390/biology14060590/s1, Figure S1: The response curves of *Sargassum* to the selected oceanographic environmental variables.; Figure S2: The distribution of the predicted habitat suitability index (HSI) of *Sargassum* based on the Maximum Entropy (MAXENT) model for the 2030s (2030–2040).; Figure S3: The distribution of the predicted habitat suitability index (HSI) of *Sargassum* based on the Maximum Entropy (MAXENT) model for the 2060s (2060–2070).; Figure S4: The changes in suitable habitats for *Sargassum* under the climate change scenarios in the 2030s (2030–2040) compared to the present (2000–2020).; Figure S5: The changes in suitable habitats for *Sargassum* under the climate change scenarios in the 2060s (2060–2070) compared to the present (2000–2020).; Figure S6: The richness of *Sargassum* under future climate change scenarios in the 2030s (2030–2040) and 2060s (2060–2070).; Table S1: Latitude and longitude coordinates of 591 occurrence records of four *Sargassum* species in South Korea.; Table S2: Oceanographic environmental variables, units, and original resolution downloaded from Bio-Oracle v3.0.

## Abbreviations

The following abbreviations are used in this manuscript:

HSI	Habitat suitability index
MAXENT	Maximum Entropy
SDM	Species distribution model
SSP	Shared Socioeconomic Pathway
MPA	Marine protected area
