# Supplementary material for: Spatio-Temporal Projections of the Distribution of the Canopy-Forming Algae Sargassum in the Western North Pacific Under Climate Change Scenarios Using the MAXENT Model
Source: Biology (Basel). 2025 May 22;14(6):590. doi: 10.3390/biology14060590 (PMC12189625; doi:10.3390/biology14060590)
Supplement: Supplementary file 1 [file biology-14-00590-s001.zip › Supplementary Materials (Figures S1-S6 and Table S2).pdf]

Supplementary Materials

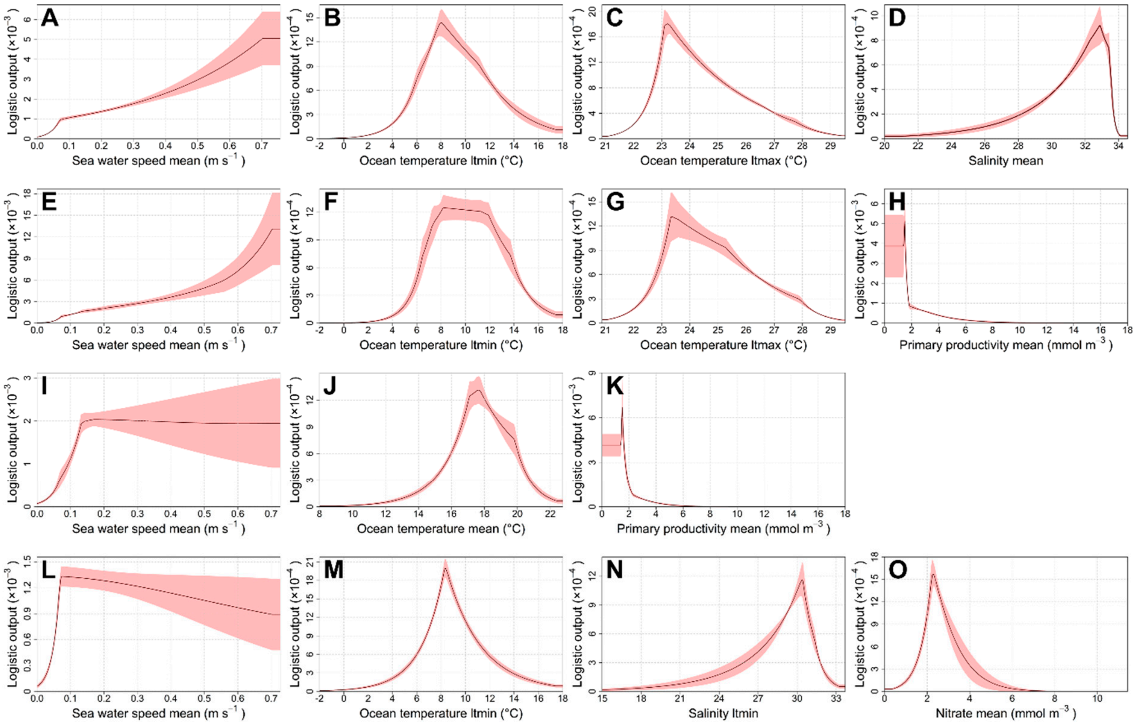

Figure S1. The response curves of *Sargassum* to the selected oceanographic environmental variables. A–D: the mean of current velocity (m s^{-1}), ltmin of water temperature (°C), ltmax of water temperature (°C), and mean of salinity for *S. horneri*. E–H: the mean of current velocity, ltmin of water temperature, ltmax of water temperature, and mean of primary productivity (mmol m^{-3}) for *S. macrocarpum*. I–K: the mean of current velocity, mean of water temperature (°C), and mean of primary productivity for *S. patens*. L–O: the mean of current velocity, ltmin of water temperature, ltmin of salinity, and mean of nitrate concentration (mmol m^{-3}) for *S. piluliferum*.

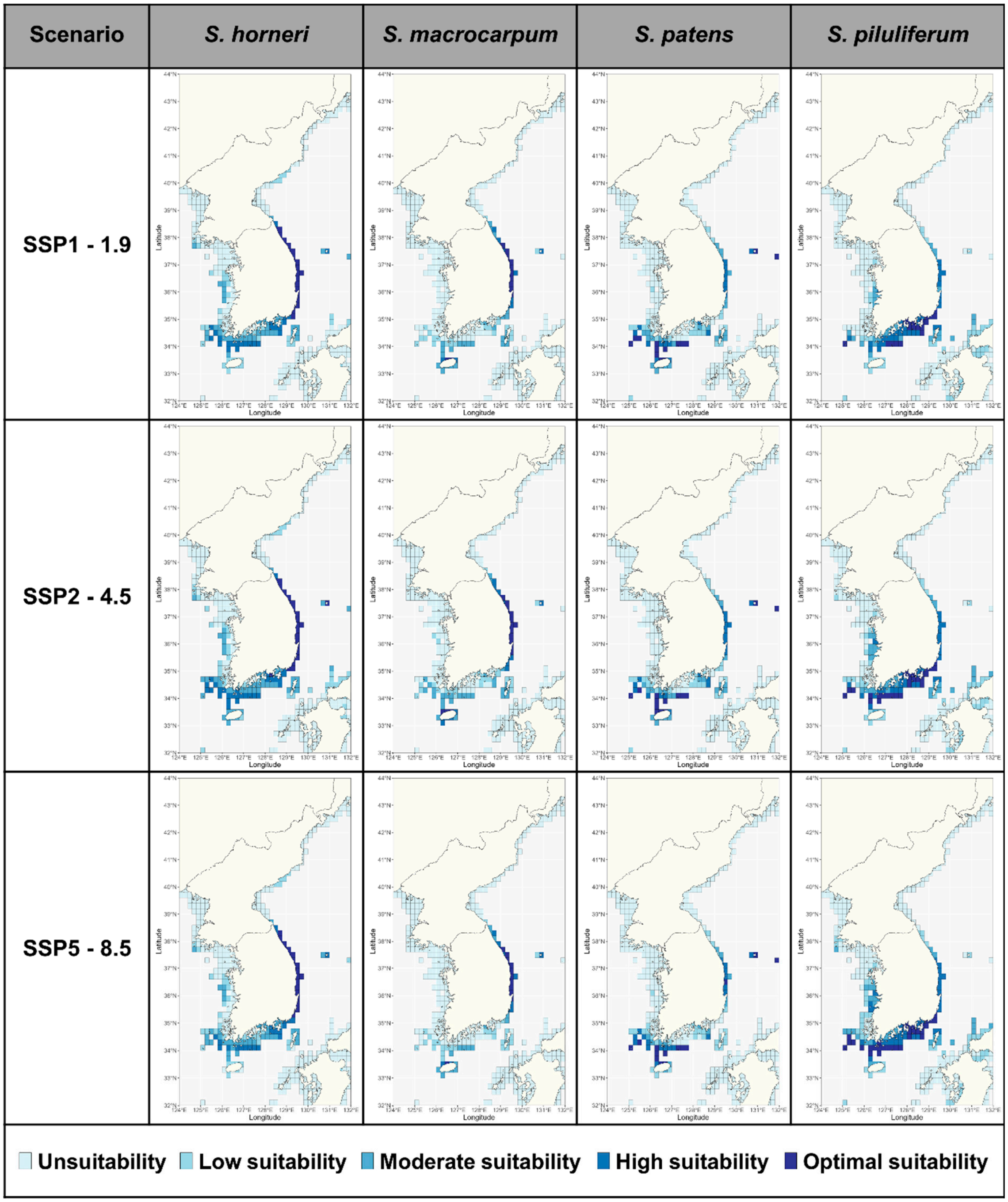

Figure S2. The distribution of the predicted habitat suitability index (HSI) of *Sargassum* based on the Maximum Entropy (MAXENT) model for the 2030s (2030–2040). The HSIs are defined by MAXENT, which assigns a MAXENT result of 0–0.2 to a HSI of 'Unsuitability', 0.2–0.4 to 'Low suitability', 0.4–0.6 to 'Moderate suitability', 0.6–0.8 to 'High suitability', and 0.8–1 to 'Optimal suitability'.

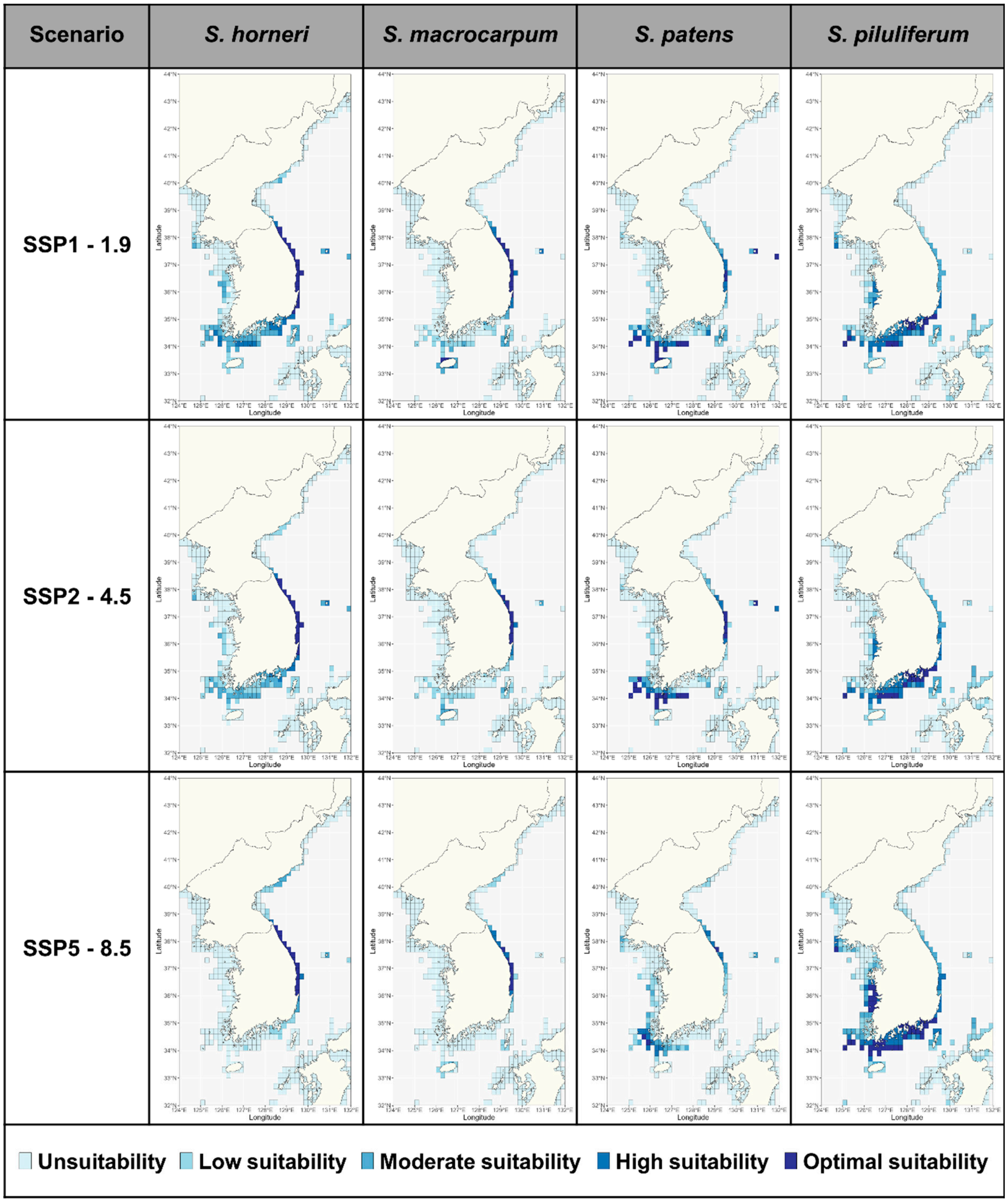

Figure S3. The distribution of the predicted habitat suitability index (HSI) of *Sargassum* based on the Maximum Entropy (MAXENT) model for the 2060s (2060–2070). The HSIs are defined by MAXENT, which assigns a MAXENT result of 0–0.2 to a HSI of 'Unsuitability', 0.2–0.4 to 'Low suitability', 0.4–0.6 to 'Moderate suitability', 0.6–0.8 to 'High suitability', and 0.8–1 to 'Optimal suitability'.

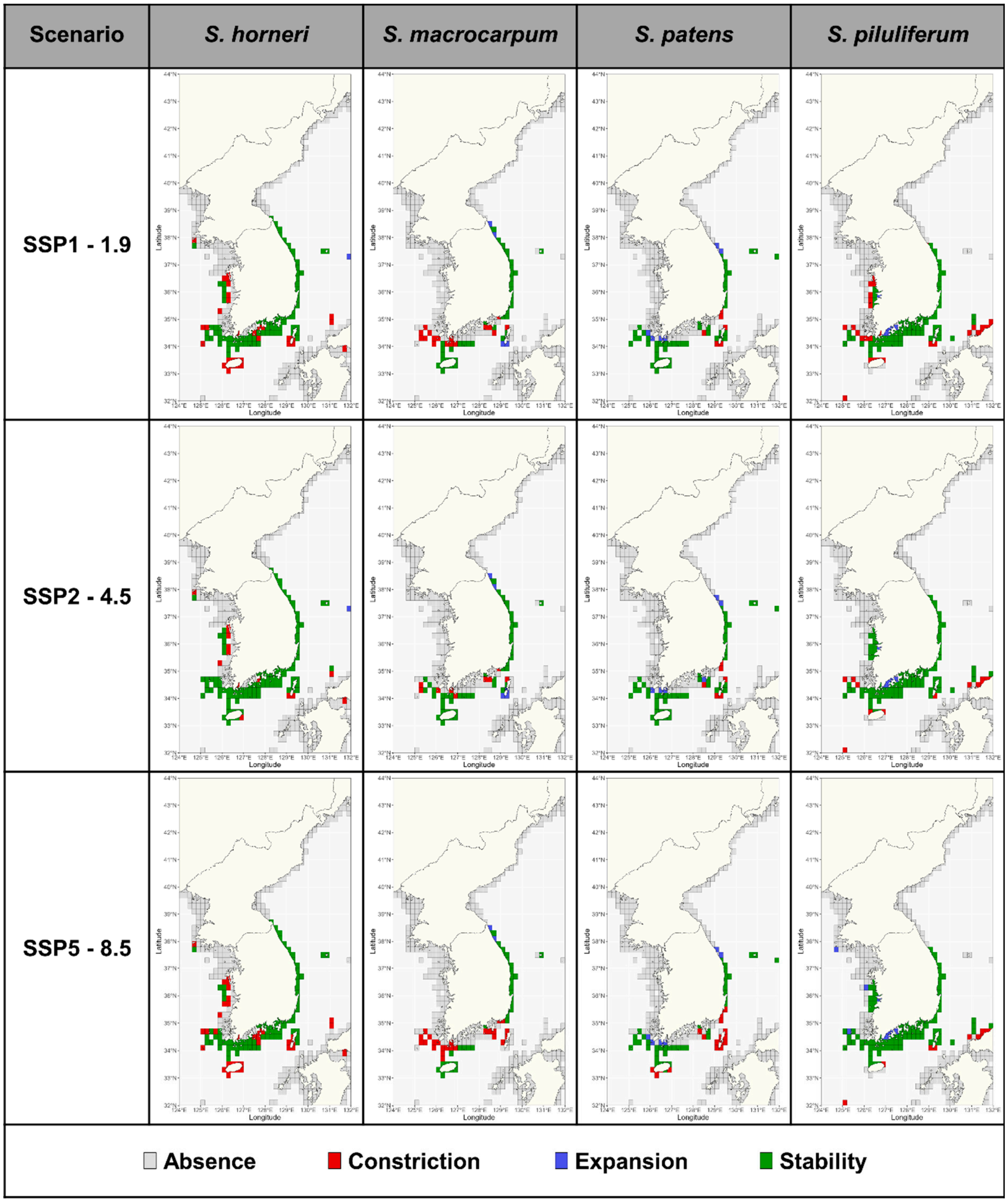

Figure S4. The changes in suitable habitats for *Sargassum* under the climate change scenarios in the 2030s (2030–2040) compared to the present (2000–2020). 'Absence' means unsuitable habitat (MAXENT < 0.4) in the present and future, 'Constriction' means changes from suitable habitat (MAXENT ≥ 0.4) to unsuitable habitat, 'Expansion' means changes from unsuitable habitat to suitable habitat, and 'Stability' means the habitat is suitable in both periods.

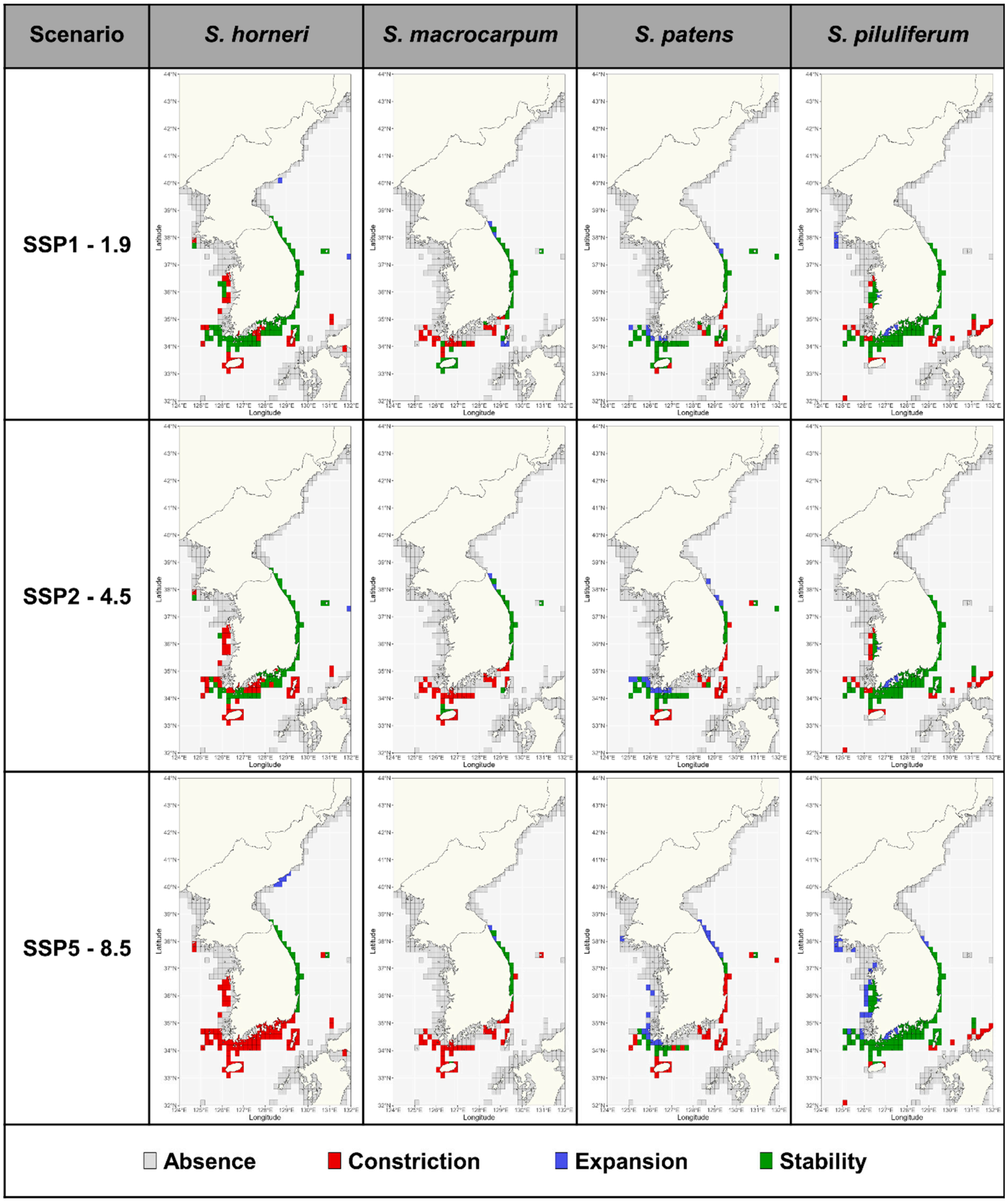

Figure S5. The changes in suitable habitats for *Sargassum* under the climate change scenarios in the 2060s (2060–2070) compared to the present (2000–2020). 'Absence' means unsuitable habitat (MAXENT < 0.4) in the present and future, 'Constriction' means changes from suitable habitat (MAXENT ≥ 0.4) to unsuitable habitat, 'Expansion' means changes from unsuitable habitat to suitable habitat, and 'Stability' means the habitat is suitable in both periods.

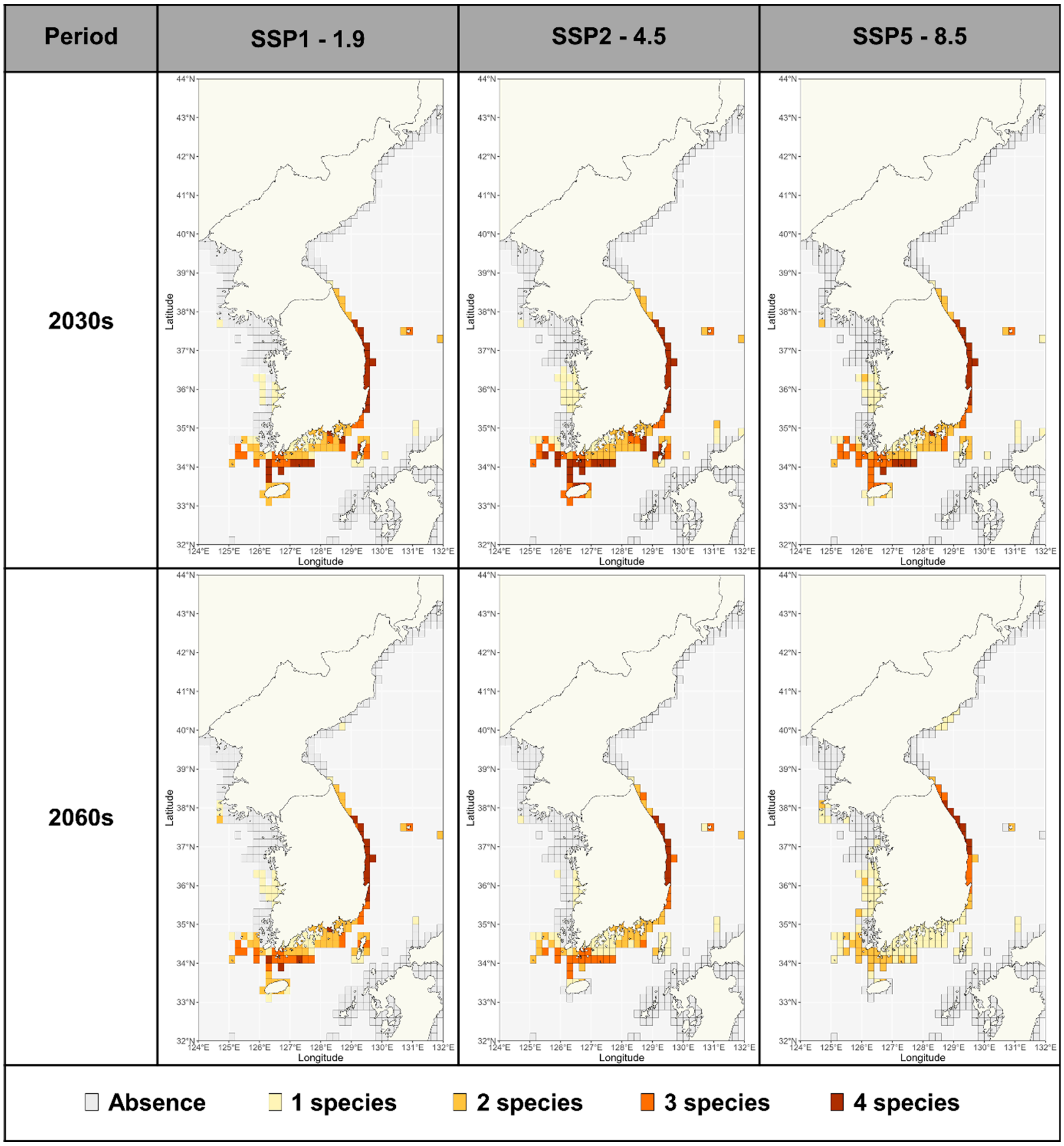

Figure S6. The richness of *Sargassum* under future climate change scenarios in the 2030s (2030–2040) and 2060s (2060–2070). The numbers represent the number of species that deem the habitat suitable (MAXENT ≥ 0.4), and 'Absence' means that all species deem the habitat unsuitable.

Table S1. Latitude and longitude coordinates of 591 occurrence records of four Sargassum species in South Korea.

(Attached Excel file)

Table S2. Oceanographic environmental variables, units, and original resolution downloaded from Bio-Oracle v3.0. Only the variables highlighted in bold were selected in this study.

Variable	Units	Original Resolution
**Ocean temperature (Ltmax)**	°C	0.05 degree
**Ocean temperature (Ltmin)**	°C	0.05 degree
**Ocean temperature (Mean)**	°C	0.05 degree
Salinity (Ltmax)	-	0.05 degree
**Salinity (Ltmin)**	-	0.05 degree
**Salinity (Mean)**	-	0.05 degree
**Sea water velocity (Mean)**	$m\ s^{-1}$	0.05 degree
pH (Mean)	-	0.05 degree
**Primary productivity (Mean)**	$mmol\ m^{-3}$	0.05 degree
**Nitrate (Mean)**	$mmol\ m^{-3}$	0.05 degree
Phosphate (Mean)	$mmol\ m^{-3}$	0.05 degree
